# Exosomal miR-301a-3p from esophageal squamous cell carcinoma cells promotes angiogenesis by inducing M2 polarization of macrophages via the PTEN/PI3K/AKT signaling pathway

**DOI:** 10.1186/s12935-022-02570-6

**Published:** 2022-04-18

**Authors:** Yuwei Shou, Xiaoqian Wang, Chao Chen, Yinghao Liang, Chenbo Yang, Qiankun Xiao, Hui Li, Shuaiyuan Wang, Jiao Shu, Xiangyu Tian, Kuisheng Chen

**Affiliations:** 1 Department of Pathology, The First Affiliated Hospital of Zhengzhou University, Zhengzhou, 450000 China; 2 Academy of Medical Sciences, Zhengzhou University, Zhengzhou, 450000 China; 3 Henan Key Laboratory of Tumor Pathology, Zhengzhou University, Zhengzhou, 450000 China

**Keywords:** Exosomes, miR-301a-3p, Macrophage polarization, PI3K/AKT signaling pathway, Angiogenesis, Esophageal squamous cell carcinoma

## Abstract

**Background:**

Growing evidence has indicated that tumor-associated macrophages (TAMs) promote tumor angiogenesis. However, the mechanisms underlying the pro-angiogenic switch of TAMs remains unclear. Here, we examined how exosomal miR-301a-3p secreted by esophageal squamous cell carcinoma (ESCC) cells triggers the pro-angiogenic switch of TAMs.

**Methods:**

We quantified miR-301a-3p levels in ESCC tumors using qRT-PCR. Macrophage phenotypes were identified using flow cytometry and qRT-PCR. The pro-angiogenic ability of TAMs was measured using the CCK-8 assay, scratch assay, Transwell migration and invasion assay, and tube formation assay. The mechanism by which exosomal miR-301a-3p secreted by ESCC cells triggers the pro-angiogenic switch of TAMs was elucidated using western blots, qRT-PCR, and a dual-luciferase reporter assay.

**Results:**

We observed anomalous miR-301a-3p overexpression in ESCC tumor tissues and cell lines. Then, we verified that ESCC-derived exosomes promoted angiogenesis by inducing macrophage polarization into M2 type, and exosomal miR-301a-3p secreted by ESCC cells was responsible for this effect. Finally, we discovered that exosomal miR-301a-3p promoted M2 macrophage polarization via the inhibition of PTEN and activation of the PI3K/AKT signaling pathway, subsequently promoting angiogenesis via the secretion of VEGFA and MMP9.

**Conclusion:**

The pro-angiogenic switch of TAMs is triggered by exosomal miR-301a-3p secreted from ESCC cells via the PTEN/PI3K/AKT signaling pathway. Although tumor angiogenesis can be regulated by a wide range of factors, exosomal miR-301a-3p could hold promise as a novel anti-angiogenesis target for ESCC treatment.

## Introduction

In the past few decades, surgical resection, chemotherapy, targeted therapy, immunotherapy, and radiotherapy have partially improved therapeutic efficacy in cases of esophageal cancer. However, this malignancy still has the seventh-highest incidence rate and sixth-highest fatality rate among all malignancies worldwide [1]. There are two predominant histological subtypes of esophageal cancer, which differ in epidemiology and pathology: esophageal adenocarcinoma (ECA) and esophageal squamous cell carcinoma (ESCC). ESCC accounts for a large majority of all esophageal cancer cases worldwide [2]. As its early symptoms are not obvious, most patients are diagnosed with the disease at the middle or advanced stage. Therefore, the prognosis is unfavorable, and the overall 5-year survival rate is below 20% [3].

Macrophages are the most common invasive immune effector cells in the tumor microenvironment (TME). They have significant plasticity, and can change their phenotypes and play diverse roles according to the surrounding signals [4]. Macrophages have two primary phenotypes: the M1 and M2 phenotypes. The M1 phenotype occurs in macrophages that are activated in a typical manner and is induced by lipopolysaccharide (LPS) or interferon-γ (IFN-γ). High levels of interleukin (IL)-12 production and a strong ability to present antigens are characteristic of M1-type macrophages. Accordingly, these cells are commonly considered efficient effector cells that regulate adaptive Th1 immunity and can kill microorganisms and tumor cells, while also producing a large number of pro-inflammatory cytokines to promote inflammation. In contrast, the M2 phenotype occurs in macrophages undergoing alternative activation and can be induced by IL-4, IL-10, CSF-1, or transforming growth factor β (TGF-β). M2-type macrophages show high levels of anti-inflammatory cytokines (e.g., IL-10). Hence, it is believed that they regulate adaptive Th2 immunity, promote angiogenesis and tissue repair, have anti-inflammatory effects, and are conducive to tumor growth [5]. Tumor associated macrophages (TAMs) are typically M2-type macrophages that play important roles in the TME. In addition, they are the main producers of pro-angiogenic factors in several types of tumors. They promote tumor angiogenesis by secreting a variety of pro-angiogenic cytokines, including vascular endothelial growth factor A (VEGFA), platelet-derived growth factor β, angiogenin, placental growth factor, TGF-β, and matrix metalloproteinases (MMPs) [6]. Nevertheless, how tumor cells transform macrophages into anti-inflammatory M2-type macrophages is still not clearly known.

Exosomes are extracellular vesicles derived from cells and have diameters of 30–200 nm. They can transfer intracellular goods including microRNAs (miRNAs), messenger RNAs (mRNAs), and proteins to recipient cells, participate in intercellular communication, and contribute to tumor growth, metastasis, and angiogenesis [7]. miRNAs, which are approximately 20–24 nucleotides in length, are small endogenous non-coding RNA molecules. miRNAs are significant regulators of gene expression and participate in regulating translation by acting on the 3′ untranslated regions (3′UTR) of specific mRNAs [8]. Exosomal miRNAs from cancerous cells play significant roles in macrophage polarization [9]. However, how these miRNAs enable TAMs to exert pro-angiogenic effects via intercellular crosstalk remains unclear.

Growing tumors critically depend on the oxygen and nutrition delivered by the tumor angiogenic system for survival. Thus, tumor angiogenesis is considered a crucial pathologic feature of cancer that has a close association with tumor growth and progression [10]. In 1996, Hanahan et al. proposed the concept of the "angiogenic switch" and the angiogenesis switch balance hypothesis. They suggested that tumor angiogenesis depends on the balance regulated by the pro- and anti-angiogenic factors secreted by tumor cells or surrounding stromal cells, and an increase in promoting factors or decrease in inhibitory factors would shift the balance towards a pro-angiogenic state [11]. The PI3K/AKT signaling pathway controls cell growth and survival, and its continuous activation is closely related to cellular transformation, tumorigenesis, tumor metastasis, and angiogenesis [12]. Studies have verified that the PI3K/AKT signaling pathway participates in tumor angiogenesis by regulating several pro-angiogenic cytokines, such as VEGFA and MMP9 [13]. PTEN is a phosphatase that can dephosphorylate PIP3 into PIP2 and directly curb the oncogenic PI3K/AKT signaling pathway [14]. Accordingly, loss of PTEN tumor suppressor function is one of the most common events observed in the pathogenesis of various malignant tumors. However, whether PTEN and the PI3K/AKT pathway participate in triggering the pro-angiogenic switch of TAMs and whether exosomal miRNAs from cancerous cells can control these two regulators remain to be elucidated.

Therefore, here, we examined the mechanism via which the pro-angiogenic switch of TAMs is triggered in cases of ESCC, specifically focusing on the interplay among exosomal miR-301a-3p, PTEN, and the PI3K/AKT signaling pathway.

## Materials and methods

### Collection of clinical tissue samples and ethics statement

We collected 20 pairs of fresh ESCC samples and surrounding normal esophageal mucosal samples from patients diagnosed with ESCC in the First Affiliated Hospital of Zhengzhou University (November 2020 to July 2021). All patients provided signed informed consent, and the ethics committee of the First Affiliated Hospital of Zhengzhou University provided approval for the study. Among the 20 patients, 13 were male and 7 were female, aged 51–79 years; invasion to mucosa or muscle layer in 8 cases, and to the whole esophageal layer in 12 cases; there were 10 cases without lymph node metastasis and 10 cases with lymph node metastasis. According to the 8th TNM staging criteria for esophageal cancer jointly released by THE International Union against Cancer (UICC) and the American Cancer Federation (AJCC) in 2017, there were 11 cases of stages I–II and 9 cases of stages III–IV.

### miRNA target prediction

The analysis of miRNAs predicted to target the *PTEN* gene was performed using Target Scan (http://www.targetscan.org/vert_72/), miRDB (http://mirdb.org), and miDIP (http://ophid.utoronto.ca/mirDIP/index.jsp#r). ESCC-related miRNA expression data were obtained from the GSE97051 dataset in the GEO DataSets collection (https://www.ncbi.nlm.nih.gov/gds/?term=).

### Cell culture

EC9706 cells (ESCC cells; obtained from the Institute of Oncology, Chinese Medical College, Shenyang, China) and Human umbilical vein endothelial cells (HUVECs; obtained from Beina, Suzhou, China) were grown in Dulbecco's Modified Eagle Medium (DMEM) (GIBCO, New York, USA) supplemented with 10% fetal bovine serum (FBS, GIBCO) and 1% penicillin–streptomycin (Beyotime, Shanghai, China). THP-1 cells (Baikang, Shanghai, China), a line of human monocytes, were cultured in RPMI-1640 medium (GIBCO) containing 10% FBS and 1% penicillin–streptomycin. All cells were incubated in 5% CO_2_ at 37 °C.

THP-1 cells were incubated with 100 ng/mL phorbol 12-myristate 13-acetate (PMA, PeproTech, USA) for 48 h for macrophage induction. miR-301a-3p was overexpressed or inhibited in EC9706 cells via the stable transfection of lentiviral constructs using the manufacturer’s instructions. The miR-301a-3p mimic, miR-301a-3p inhibitor, and corresponding negative control vectors (miR-NC) were synthetized by GenePharma (Shanghai, China).

### Cell activation

After that HUVECs cells are confirmed to be attached to the wall after 6 h, the medium is changed to the EC9706 cell supernatant for cell activation. HUVECs in the exponential growth phase were inoculated in 6-well plates at a density of 5 × 10^4^/per well, and 6 groups were set up: NC group, 20% (v/v) supernatant group, 40% (v/v) supernatant group, 50% (v/v) supernatant group, 60% (v/v) supernatant group and 80% (v/v) supernatant group. The endothelial cells were cultured for 48 h using different concentrations of supernatant to screen the optimal activation for the induction conditions of TECs. After TECs were screened, EC9706 supernatant was discarded and replaced with normal medium for subsequent experiments.

### Isolation and identification of exosomes

At 60–70% confluence, ESCC cells were grown in serum-free DMEM for 48 h. We obtained supernatant from the cell culture and filtered it using 0.22-μm filters (Millipore, Darmstadt, Germany). The filtered supernatant was subjected to centrifugation (3000×*g*, 15 min) to discard any remaining cells and cell fragments. Subsequently, it was incubated with the Exo-Quick exosome precipitation solution (SBI, California, USA) overnight at 4 °C based on the manufacturer’s protocol. After 12 h of incubation, the mixture underwent centrifugation (1500×*g*, 30 min), and the obtained particles were resuspended in 100 μL PBS after discarding the supernatant.

A transmission electron microscope (TEM, Phillips, Eindhoven, Netherlands) was used to observe exosomes after they were placed on carbon-coated grids and treated with 2% uranyl acetate for 3 min. The sizes and concentrations of the particles were examined with a nanoparticle analyzer (Nanosight, Great Malvern, UK) for nanoparticle tracking analysis (NTA). Specific markers of exosomes (CD81 and CD63) were examined with western blots.

### Exosome uptake assay

Exosomes were labeled using the PKH26 Red Fluorescent Cell Linker Kit (Sigma-Aldrich, St. Louis, USA). First, 100 μL exosome suspensions were mixed with 100 μL of Diluent C to obtain Diluent A. Diluent B was prepared by mixing the PKH26 ethanolic dye with 100 μL of Diluent C. After mixing Diluent A with Diluent B and incubating the mixture for 5 min, 200 μL of 5% bovine serum albumin (BSA, GIBCO) solution was added to stop the staining reaction. Then, PKH26-labeled exosomes were isolated by incubating the above solution with the Exo-Quick exosome precipitation solution (SBI) for 12 h at 4 °C based on the manufacturer’s protocol, and then performing centrifugation at 1500×*g* for 30 min. After co-culturing exosomes with macrophages for 24 h, the macrophages were fixed with 4% paraformaldehyde (Solarbio) for 20 min and their nuclei were stained with 4′,6-diamidino-2-phenylindole (DAPI, Solarbio). Fluorescence images showing exosome uptake by macrophages were obtained using laser scanning confocal microscopy (ZEISS, Oberkohen, Germany).

### Dual-luciferase reporter assay

Lipofectamine 2000 (Invitrogen, USA) was employed for co-transfecting the PTEN-3'-UTR WT (GenePharma) and PTEN-3'-UTR MUT constructs with a miR-301a-3p mimic or miR-NC construct, respectively, into 293T cells. 48 h later, a dual-luciferase reporter assay system was used to detect the luciferase activity (Promega, WI, USA).

### RNA extraction and quantitative real‑time polymerase chain reaction (qRT-PCR)

Total RNA was extracted from cells and exosomes using the Trizol reagent (Beyotime, Shanghai, China). For the analysis of miRNAs, the obtained RNA was reverse transcribed into complementary DNA (cDNA) using the miRNA 1st strand cDNA synthesis Kit (Vazyme, Nanjing, China), following which the miRNA universal SYBR qPCR Master Mix (Vazyme) was used for qPCR. For mRNA level analysis, the PrimeScript™ RT reagents Kit (Takara, Japan) was used to synthesize cDNA from the extracted total RNA. Subsequently, the TB Green Premix Ex Taq II (Takara, Japan) system was used for qPCR. U6 and GAPDH were chosen as endogenous miRNA and mRNA controls for the analysis, respectively. Primer synthesis was performed by SANGON (Shanghai, China) (Table 1). The 2^−ΔΔCt^ method was applied for quantifying relative miRNA and mRNA levels.

**Table 1:** Primer sequences for qRT-PCR

Gene	Primer sequence
Mir-301a-3p	F: CGCGCAGTGCAATAGTATGT
	R: AGTGCAGGGTCCGAGGTATT
PTEN	F: TGACAGTGCGAGACTCCATC
	R: GGACGAACTGGTGTAATGATATG
PI3K	F: GTCCTATTGTCGTGCATGTGG
	R: TGGGTTCTCCCAATTCAACC
AKT	F: TTCTATGGCGCTGAGATTGTGT
	R: GCCGTAGTCATTGTCCTCCAG
CD206	F: GGGTTGCTATCACTCTCTATGC
	R: TTTCTTGTCTGTTGCCGTAGTT
IL-10	F: GACTTTAAGGGTTACCTGGGTTG
	R: TCACATGCGCCTTGATGTCTG
CD163	F: TTTGTCAACTTGAGTCCCTTCAC
	R: TCCCGCTACACTTGTTTTCAC
Arginase-1	F: GGTTTTTGTTGTTGCGGTGTTC
	R: CTGGGATACTGATGGTGGGATGT
VEGFA	F: GCACATAGAGAGAATGAGCTTCC
	R: CTCCGCTCTGAACAAGGCT
MMP9	F: GCAGAGGCATACTTGTACCG
	R: TGATGTTATGATGGTCCCACTTG
U6	F: CTCGCTTCGGCAGCACA
	R: AACGCTTCACGAATTTGCGT
GAPDH	F: GAAGGTGAAGGTCGGAGTCA
	R: AATGAAGGGGTCATTGATGG

### Protein extraction and western blot

Total protein was extracted from cells and exosomes using the RIPA lysis buffer (Solarbio), which contains protease inhibitors (Vazyme) and phosphatase inhibitors (Vazyme). Sample protein levels were examined with the BCA Protein Assay Kit (Thermo Fisher, Waltham, USA). Protein levels in the experimental and control groups were adjusted to equivalent levels by adding RIPA lysis buffer and 5 × loading buffer (Solarbio). Next, the protein samples were heated at 100 °C for 10 min, following which 10% SDS–PAGE electrophoresis was performed using 20 mg of samples (30 min, 80 V and then 1 h, 110 V). Proteins were then transferred to poly-vinylidene difluoride membranes (Millipore, USA) (2 h, 300 mA), which were subsequently blocked in 5% bovine serum albumin (Solarbio) for 2 h. Following this, the membranes were incubated with primary antibodies (Abcam, Cambridge, UK) overnight at 4 °C. Then, they were incubated with horseradish peroxidase-conjugated secondary antibodies (Abcam) for 2 h. Enhanced chemiluminescence reagents (Thermo Fisher) were used to detect protein expression.

### Flow cytometry

Antibodies against surface markers of M2 macrophages (CD206-FITC; Biolegend, California, USA) and surface markers of macrophages (CD68-APC; Biolegend) were used to label macrophages for 30 min at 4 °C. Subsequently, these different cell groups were detected using a BD FACSAria III flow cytometer (BD Biosciences, Franklin lakes, USA).

### Cell counting kit-8 (CCK-8) assay

After planting 3 × 10^3^ cells in each well, 10 μL of the CCK-8 solution (Shangbao Biology, Shanghai, China) was added, and cells were incubated at 37 °C for 2 h. Finally, optical density (OD) was examined at 450 nm with a microplate reader (Molecular Devices, San Francisco, USA).

### Scratch assay

After planting cells in a 6-well plate and controlling cell density at 5 × 10^5^ cells per well, scratches were made using a sterilized 100-μL pipette tip (same tip for all experiments). Floating cells were discarded after cleaning with PBS. Next, the scratches were recorded at 0 and 24 h. The wound width proportion was the ratio between the migrated distance and the total width of the wound.

### Transwell migration and invasion assay

Transwell migration assay: 200 μL FBS-free DMEM (with 2 × 10^5^ cells) was transferred to one chamber of a 6-well plate after 500 μL DMEM with 20% FBS was added to the well outside the chamber.

Transwell invasion assay: 100 μL Matrigel was melted in one chamber of a 6-well plate before 200 μL FBS-free DMEM (with 5 × 10^5^ cells) and 500 μL DMEM with 20% FBS was transferred to the chamber and the well outside the chamber, respectively.

In both assays, after incubation at 4 °C for 16 h, cells were fixed with 4% paraformaldehyde (Solarbio) for 20 min, and crystal violet (Solarbio) was used to stain cells for 20 min. Five random fields were recorded for each chamber, and the cells that had traversed across the chamber were counted using Image J (Staten, USA).

### Tube formation assay

Matrigel (BD Biosciences, Franklin lakes, USA) was thawed at 4 °C overnight before the experiments. Subsequently, 100 μL Matrigel was added to a 24-well plate. After 40 min of incubation, 1 mL medium (containing 1 × 10^5^ cells) was added, and followed by 5–7 h of incubation at 37 °C. Subsequently, three random fields of each well were recorded, and the total branching lengths were measured using Image J.

### Enzyme-linked immunosorbent assay (ELISA)

The levels of cytokines secreted by cells were examined using ELISA kits (RayBiotech, Atlanta, USA) according to the manufacturer's protocol. OD values (450 nm) were obtained using a microplate reader.

### Statistical analyses

A minimum of three replicates was performed for each experiment, and results were presented as mean ± standard error of the mean (SEM). We analyzed between-group differences using independent t-tests. Correlation analyses were conducted with Spearman’s rank test. Relationships of clinicopathological characteristics in ESCC patients with miR-301a-3p levels were analyzed using Pearson χ^2^ test. SPSS 26.0 software (National Institutes of Health, USA) was used for all the analyses in the study, and graph preparation was performed using GraphPad Prism 8 (USA). A two-tailed *P*-value < 0.05 was considered an indicator of statistical significance.

## Results

### miR-301a-3p is enriched in ESCC tissues and cells and in ESCC-derived exosomes

**Fig. 1:**
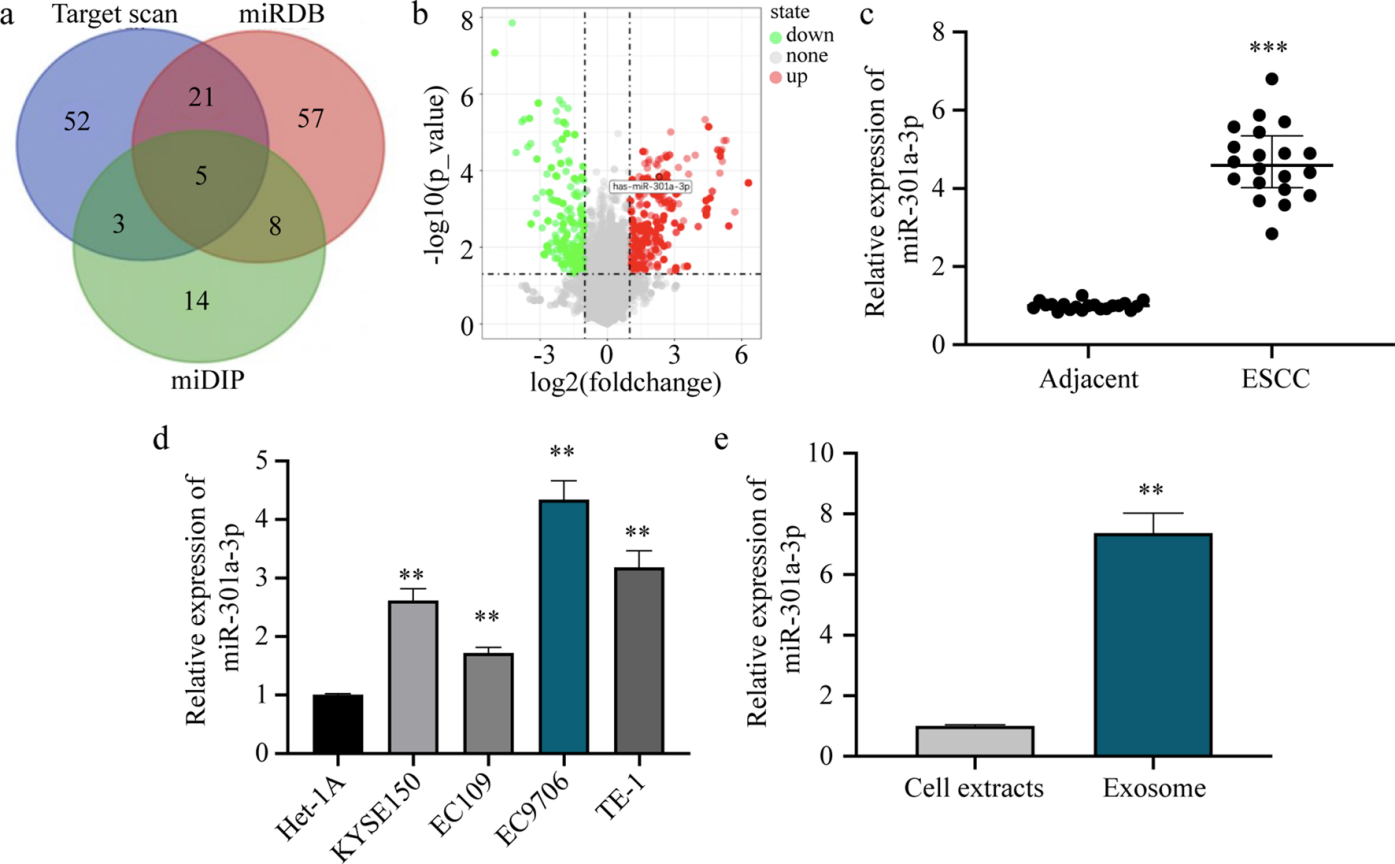
miR-301a-3p is expressed at high levels in ESCC tissues and cells and shows enrichment in ESCC-derived exosomes. **a** miRNAs targeting the *PTEN* gene were predicted using Target Scan, miRDB, and miDIP. **b** miRNA expression in ESCC tissues was illustrated using a Volcano plot. Each dot represents one miRNA. The dot representing miR-301a-3p is red in color, implying that miR-301a-3p was significantly upregulated in ESCC patients. **c–e** Quantification of miR-301a-3p expression in 20 pairs of fresh ESCC tissues and corresponding adjacent normal esophageal mucosa tissues (**c**), four ESCC cell lines KYSE150, EC109, EC9706, and TE1 and one normal esophageal mucosal cell line Het-1A (**d**), and EC9706 cells and exosomes secreted by EC9706 cells (**e**) using qRT-PCR. **p < 0.01, ***p < 0.001

PTEN is the primary regulator of the pro-angiogenic switch [15]. We attempted to identify a miRNA that can regulate this switch by directly targeting PTEN. To this end, we used three bioinformatics software, Target Scan, miRDB, and miDIP, to predict the miRNAs targeting the *PTEN* gene (Fig. 1a). Further, we obtained ESCC-related miRNA expression data (GSE97051 dataset) from the GEO DataSets and verified whether the five candidate miRNAs predicted by the software were differentially expressed in ESCC tissues. Only miR-301a-3p showed significant upregulation in ESCC tissues (Fig. 1b), indicating that miR-301a-3p might be a key regulator in ESCC.

To prove this differential expression experimentally, we performed qRT-PCR using clinical samples. miR-301a-3p was significantly upregulated in ESCC tissues in comparison with adjacent normal esophageal mucosa tissues (Fig. 1c). Further, the miR-301a-3p expression level showed a positive correlation with TNM stage (*p* = 0.005), tumor invasion (*p* = 0.008), and lymph node metastasis (*p* = 0.026), but it showed no association with age and sex (Table 2). Subsequently, expression analysis in ESCC lines (KYSE150, EC109, EC9706, and TE1) and in the normal esophageal mucosa cell line (Het-1A) revealed the highest miR-301a-3p expression in EC9706 cells. Thus, these cells were selected for subsequent experiments (Fig. 1d). Meanwhile, the miR-301a-3p level in ESCC cells was higher than that in Het-1A cells, indicating significant miR-301a-3p upregulation in ESCC cells (Fig. 1d). Hence, miR-301a-3p is enriched in ESCC tissues and cells.

**Table 2:** Relationship between miR-301a-3p expression and clinicopathologic characteristics of 20 patients with ESCC

Variables	Total	miR-301a-3p expression		
		Low	High	*P* value
Gender				
Male	13	7	6	0.961
Female	7	3	4	
Age (years)				
< 60	4	2	2	0.551
> 60	16	8	8	
Tumor diameter (cm)				
< 3	3	2	1	0.277
> 3	17	8	9	
T stage				
T1 + T2	8	7	1	0.008**
T3 + T4	12	3	9	
Lymph node metastasis				
N^−^	10	2	2	0.026*
N^+^	10	8	8	
TNM stage				
I + II	11	8	3	0.005**
III + IV	9	2	7	

In addition, qRT-PCR results demonstrated that miR-301a-3p expression was higher in exosomes secreted by EC9706 cells than in EC9706 cells, indicating that this miRNA was enriched in ESCC-derived exosomes (Fig. 1e).

### ESCC-derived exosomes induce M2 polarization of macrophages

**Fig. 2:**
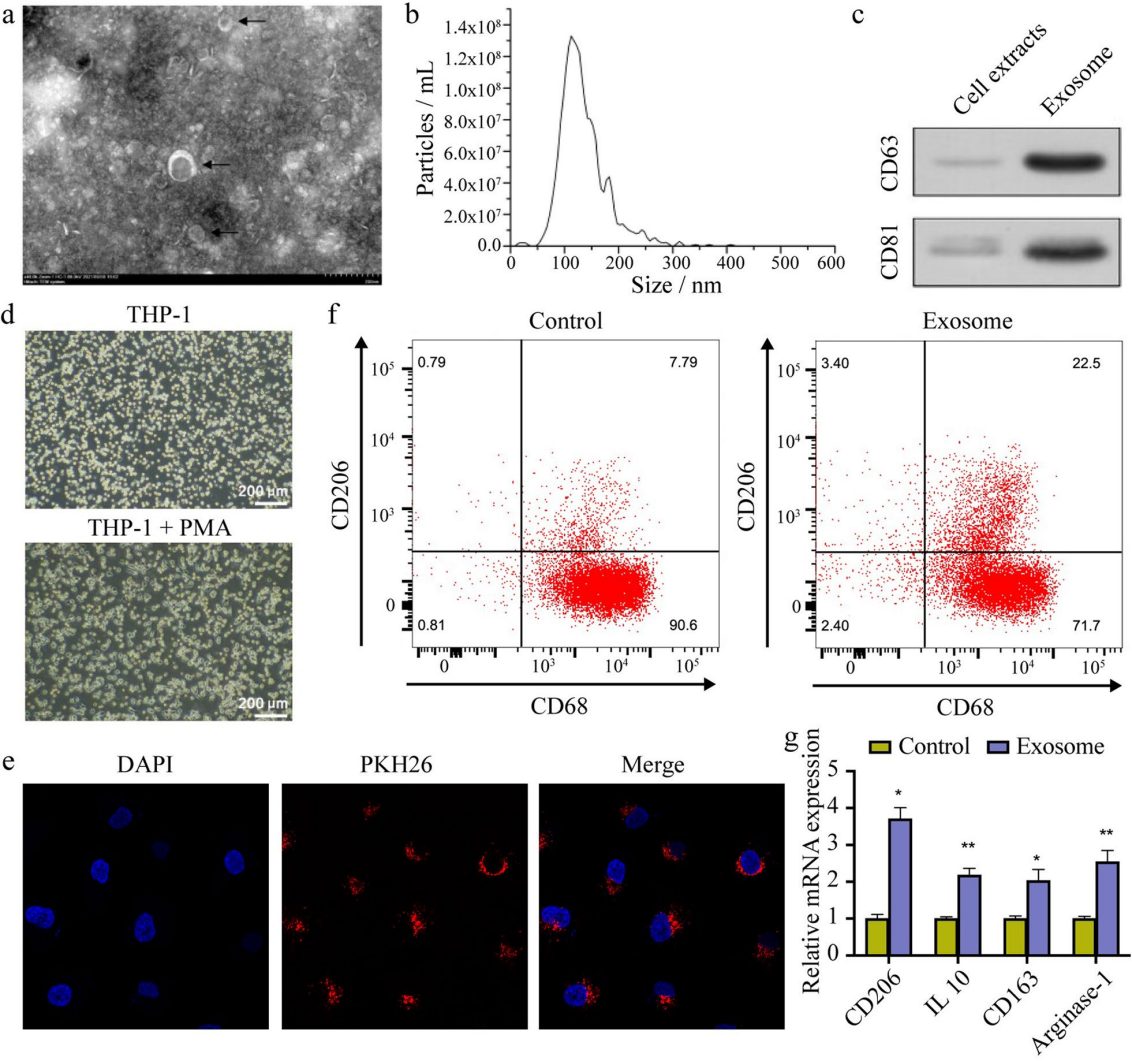
ESCC cell-derived exosomes induce M2 polarization of macrophages. **a** Exosome structure was clearly observed using transmission electron microscopy. **b** Exosomal size distribution and concentration were quantified using nanoparticle tracking analysis. **c** CD63 and CD81 expression in ESCC cell-derived exosomes was detected using western blot. **d** THP-1 cells were subjected to PMA treatment to obtain macrophages. **e** Scanning confocal microscopy images demonstrating that exosomes were internalized by macrophages. **f**, **g** Expression of M2 markers (CD206, IL-10, CD163, and Arginase-1) in macrophages incubated with exosomes was measured using flow cytometry and qRT-PCR. *p < 0.05, **p < 0.01

Exosomes were isolated from ESCC cells and identified based on three methods. As depicted in Fig. 2a and b, the typical lipid bilayer disc structures of exosomes could be clearly visualized using TEM, and their diameters were found to be about 50–150 nm using NTA. CD63 and CD81, typical markers of exosomes, were examined using western blot and were found to be enriched in ESCC-derived exosomes (Fig. 2c). Hence, the isolates were confirmed to be exosomes.

As shown in Fig. 2d, macrophages were obtained from THP-1 cells after PMA treatment. After incubating macrophages with PKH26-labeled ESCC-derived exosomes for 24 h, red fluorescence was visualized in the cytoplasm of macrophages, indicating that the exosomes were successfully internalized by macrophages (Fig. 2e).

Next, flow cytometry and qRT-PCR demonstrated that M2 markers (CD206, IL-10, CD163, and Arginase-1) showed higher levels in macrophages treated with exosomes than in those treated with PBS, suggesting that ESCC-derived exosomes can induce the M2 phenotype of macrophages (Fig. 2f and g).

### Induction and identification of tumor-associated endothelial cells

**Fig. 3:**
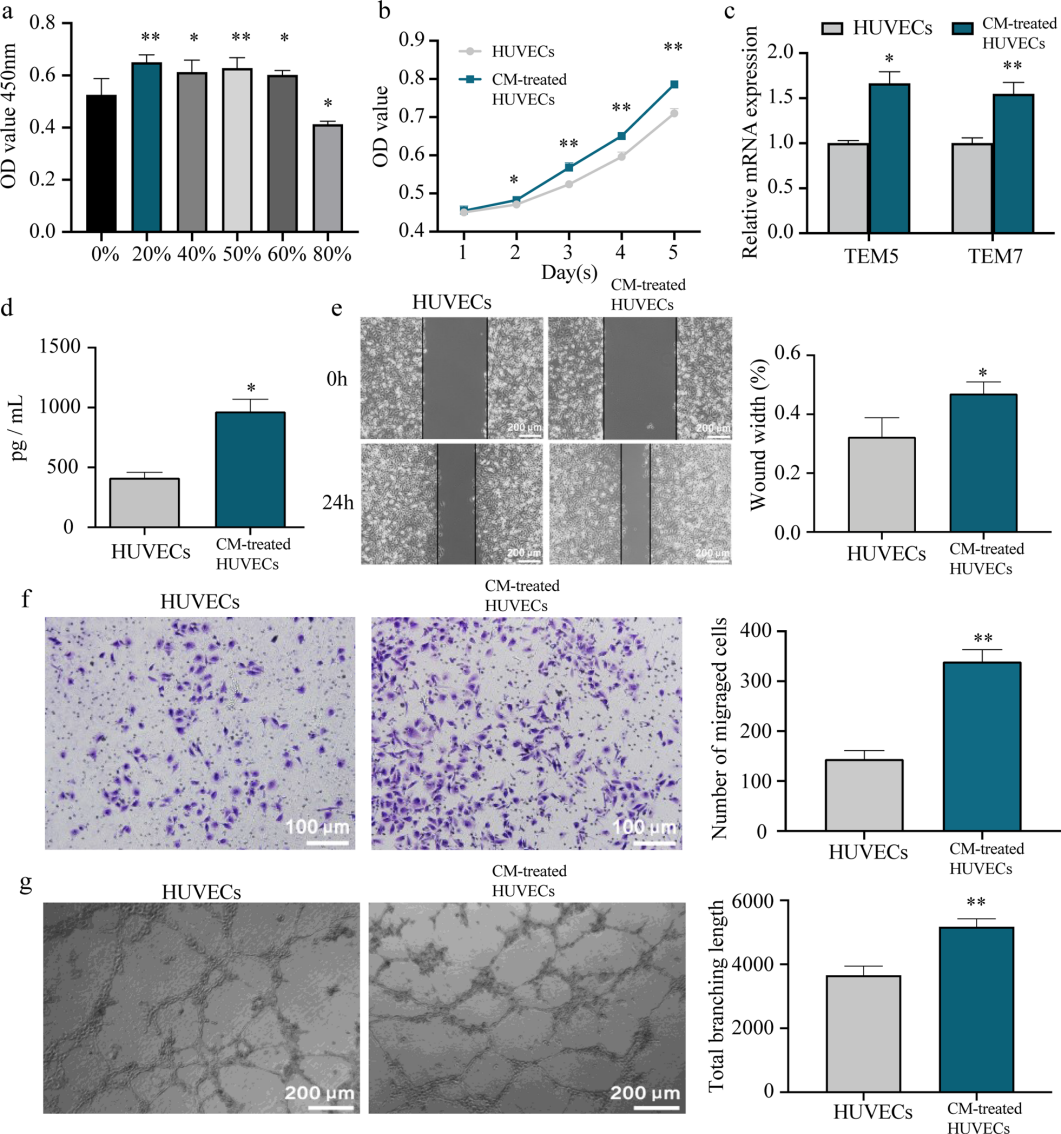
Induction and identification of tumor-associated endothelial cells. **a** Proliferation activity of HUVECs undergoing treatment with EC9706 cell supernatant (0–80%) was detected using the CCK-8 assay. **b** Proliferation ability of HUVECs co-cultured with a 20% volume fraction of EC9706 cell supernatant was detected using the CCK-8 assay. **c** Expression of tumor vascular endothelial cell markers TEM5 and TEM7 in HUVECs co-cultured with a 20% volume fraction of EC9706 cell supernatant was quantified with qRT-PCR. **d** VEGFA levels in the culture medium of HUVECs co-cultured with a 20% volume fraction of EC9706 cell supernatant was measured using ELISA. **e**, **f** Migration ability of HUVECs co-cultured with a 20% volume fraction of EC9706 cell supernatant was detected using the scratch assay and Transwell migration assay. **g** Tube formation ability of HUVECs co-cultured with a 20% volume fraction of EC9706 cell supernatant was detected using the tube formation assay. *p < 0.05, **p < 0.01

To better simulate the physiological state of endothelial cells in the ESCC TME, HUVECs were co-cultured with different concentrations of EC9706 cell supernatant as conditioned medium (CM) for 48 h, which were termed as CM-treated HUVECs, in order to allow them to transform into tumor-associated endothelial cells (TECs). Then, we used the CCK-8 assay to detect the proliferation of HUVECs co-cultured with EC9706 cell supernatant. The results demonstrated that proliferation activity was noticeably higher in HUVECs treated with medium containing a 20% volume fraction of EC9706 cell supernatant, and this was thus chosen as the optimal concentration for inducing the transformation of HUVECs into TECs (Fig. 3a). The CCK-8 assay showed that the proliferation rate of CM-treated HUVECs was enhanced at day 2 and 3 compared to HUVECs, while it was approximately parallel to HUVECs at day 4 and 5 (Fig. 3b). qRT-PCR results demonstrated that CM treatment also upregulated the tumor vascular endothelial cell markers TEM5 and TEM7 in these cells (Fig. 3c) [16, 17]. ELISA results showed that CM-treated HUVECs secreted more VEGFA than HUVECs (Fig. 3d). The scratch assay and Transwell migration assay demonstrated enhanced migration ability in CM-treated HUVECs (Fig. 3e and f), and the tube formation assay demonstrated that the total branching length of tubes formed by CM-treated HUVECs were also significantly increased (Fig. 3g). Although the CCK-8 assay could not firmly show the proliferation advantage of CM-treated HUVECs, the above multiple characterization results comprehensively confirmed the successful induction of TECs, which better simulated the existing conditions of endothelial cells in the TME and had a stronger ability to promote angiogenesis than HUVECs.

### ESCC-derived exosomes promote angiogenesis by inducing M2 polarization of macrophages

**Fig. 4:**
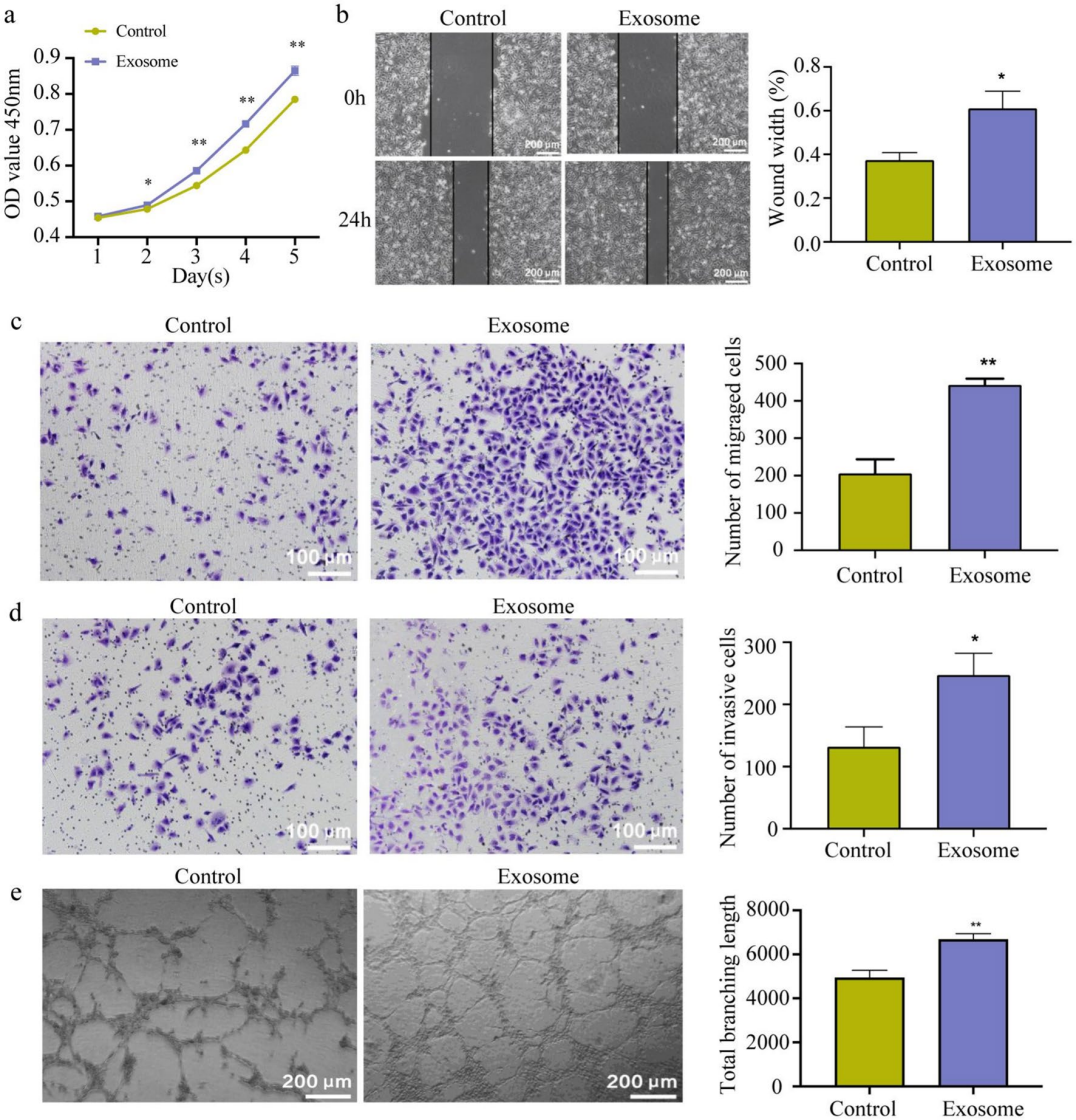
ESCC cell-derived exosomes promote angiogenesis by inducing M2 polarization of macrophages. **a** Proliferation activity of TECs was detected using the CCK-8 assay. **b–e** Migration ability, invasion ability, and tube formation ability of TECs co-cultured with the supernatant of macrophages incubated with ESCC cell-derived exosomes were detected using the scratch assay and Transwell migration assay (**b**, **c**), Transwell invasion assay (**d**), and tube formation assay (**e**), respectively. *p < 0.05, **p < 0.01

A conditioned medium system was used to explore the effect of macrophages induced by ESCC-derived exosomes on angiogenesis. The CCK-8 assay showed that supernatant from macrophages incubated with ESCC-derived exosomes promoted TEC proliferation at day 2, 3 and 4, however, did not promote TEC proliferation at day 5 (Fig. 4a). The scratch assay and Transwell migration assay suggested that supernatant from macrophages incubated with ESCC-derived exosomes promoted TEC migration (Fig. 4b and c), and the Transwell invasion assay showed that this supernatant sharply increased the number of TECs traversing through the chamber (Fig. 4d). In addition, the tube formation assay demonstrated an obvious increase in the total branching length of tubes formed by TECs after treatment with supernatant from macrophages incubated with ESCC-derived exosomes (Fig. 4e). Take these results into account comprehensively, ESCC-derived exosomes promote angiogenesis by inducing M2 polarization of macrophages.

### ESCC-derived exosomal miR-301a-3p promotes angiogenesis by inducing M2 polarization of macrophages

**Fig. 5:**
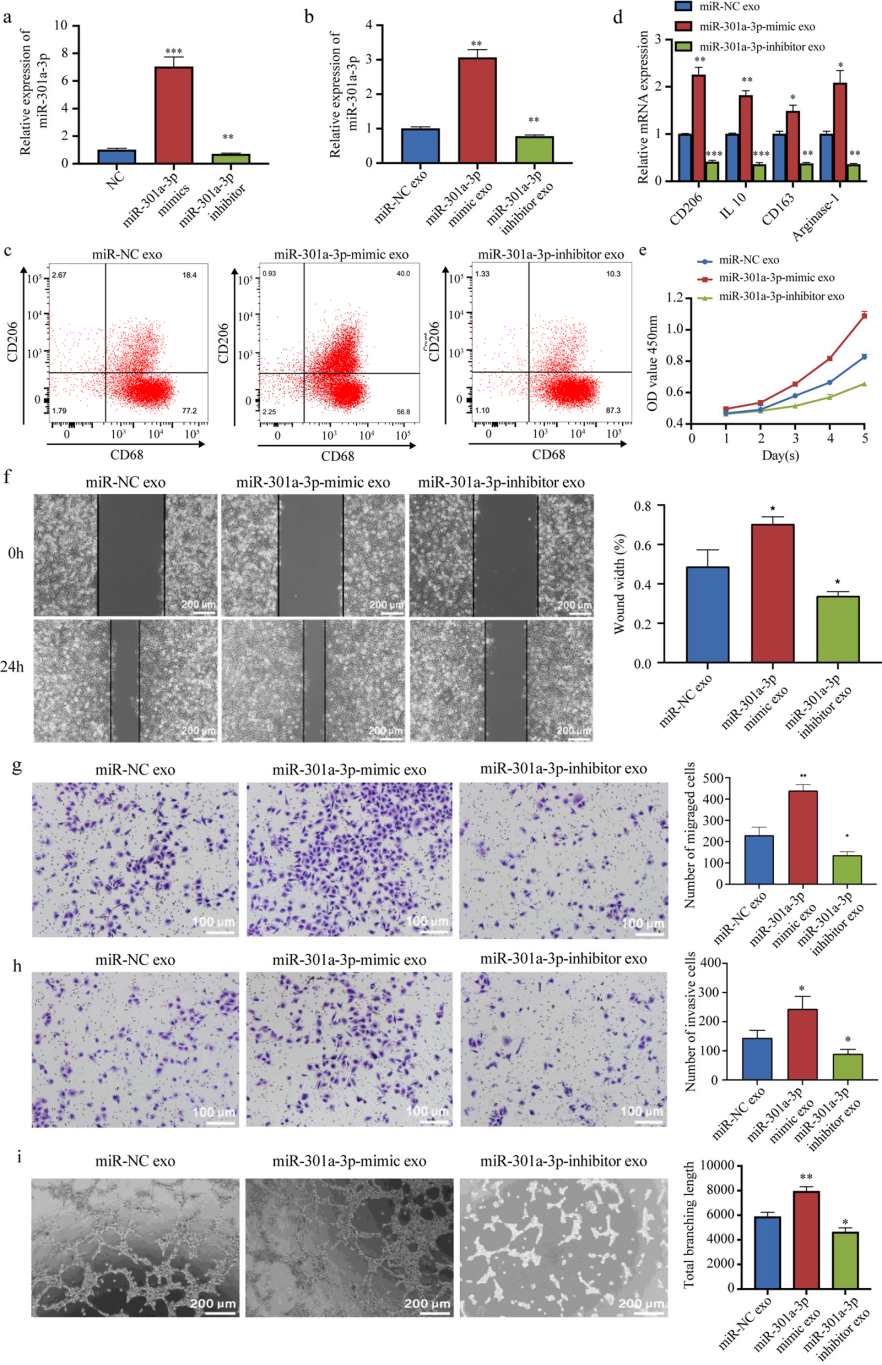
ESCC-derived exosomal miR-301a-3p promotes angiogenesis by inducing M2 macrophage polarization. **a**, **b** miR-301a-3p expression in EC9706 cells transfected with miR-NC, miR-301a-3p mimic, or miR-301a-3p inhibitor (**a**) and macrophages co-cultured with exosomes from these cells (**b**) was measured using qRT-PCR. **c**, **d** Expression of M2 markers (CD206, IL-10, CD163, and Arginase-1) in macrophages co-cultured with exosomes from EC9706 cells transfected with miR-NC, miR-301a-3p mimic, or miR-301a-3p inhibitor was measured using flow cytometry and qRT-PCR. **e–i** Proliferation activity, migration ability, invasion ability, and tube formation ability of TECs co-cultured with conditioned medium of macrophages incubated with exosomes from EC9706 cells transfected with miR-NC, miR-301a-3p mimic, or miR-301a-3p inhibitor were evaluated using the CCK-8 assay (**e**), scratch assay and Transwell migration assay (**f**, **g**), Transwell invasion assay (**h**), and the tube formation assay (**i**), respectively. *p < 0.05, **p < 0.01, ***p < 0.001

The endogenous miR-301a-3p level of EC9706 cells was altered via transfection with miR-NC, miR-301a-3p mimic, or miR-301a-3p inhibitor, and we confirmed the transfection efficiencies using qRT-PCR (Fig. 5a). Moreover, qRT-PCR confirmed that EC9706 cell-derived exosomes altered miR-301a-3p expression in macrophages (Fig. 5b), suggesting that this miRNA could be delivered to macrophages via exosomes.

Flow cytometry and qRT-PCR demonstrated the upregulation of M2 markers (CD206, IL-10, CD163, and Arginase-1) in macrophages co-cultured with exosomes from miR-301a-3p mimic-transfected EC9706 cells. In contrast, these markers were downregulated when macrophages underwent treatment with exosomes from miR-301a-3p inhibitor-transfected EC9706 cells. This confirmed that ESCC-derived exosomal miR-301a-3p can induce M2 macrophage polarization (Fig. 5c and d).

Finally, the angiogenetic effect of ESCC-derived exosomal miR-301a-3p on TECs was tested. Results showed that conditioned medium from macrophages co-cultured with exosomes derived from miR-301a-3p mimic-transfected EC9706 cells promoted TEC proliferation (Fig. 5e), migration (Fig. 5f and g), invasion (Fig. 5h), and tube formation (Fig. 5i), whereas the opposite trend was noted when miR-301a-3p inhibitor was used (Fig. 5e–i). These results demonstrated that ESCC-derived exosomal miR-301a-3p promotes angiogenesis by inducing M2 polarization of macrophages.

### ESCC-derived exosomal miR-301a-3p induces M2 macrophage polarization via the inhibition of PTEN and activation of the PI3K/AKT signaling pathway, and then promotes angiogenesis via the secretion VEGFA and MMP9

**Fig. 6:**
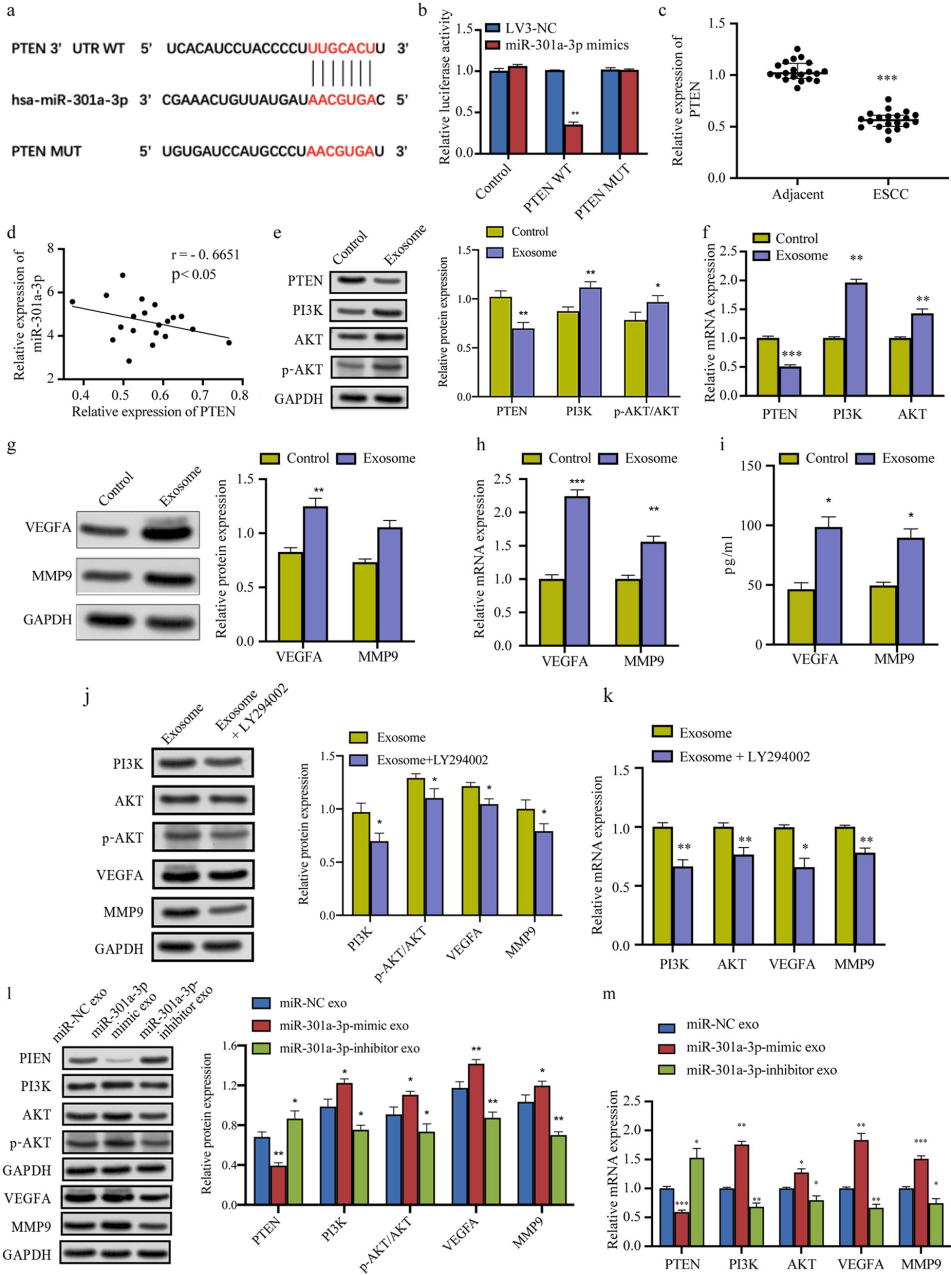
ESCC-derived exosomal miR-301a-3p inhibits *PTEN* and activates the PI3K/AKT pathway to induce the M2 polarization of macrophages, which then secrete VEGFA and MMP9 to promote angiogenesis. **a** 3′UTR sequence of *PTEN* has potential latent binding sites with the miR-301a-3p sequence. **b** Dual-luciferase reporter assay confirming the interaction of *PTEN* with miR-301a-3p. **c** *PTEN* expression in 20 pairs of fresh ESCC tissues and corresponding adjacent normal esophageal mucosa tissues was measured using qRT-PCR. **d** The relationship of miR-301a-3p expression with *PTEN* expression was analyzed with correlation analysis. **e**, **f** The expression of PTEN, PI3K, and AKT in macrophages incubated with ESCC cell-derived exosomes was measured using western blot and qRT-PCR. **g**, **h** The expression of VEGFA and MMP9 in macrophages incubated with ESCC cell-derived exosomes was measured using western blot and qRT-PCR. **i** The expression of VEGFA and MMP9 in the culture medium of macrophages incubated with ESCC cell-derived exosomes was measured using ELISA. **j**, **k** The expression of PI3K, AKT, VEGFA, and MMP9 in macrophages incubated with ESCC cell-derived exosomes and treated with the PI3K inhibitor LY294002 was measured using western blot and qRT-PCR. **l**, **m** The expression of PTEN, PI3K, AKT, VEGFA, and MMP9 in macrophages co-cultured with exosomes from EC9706 cells transfected with miR-NC, miR-301a-3p mimic, or miR-301a-3p inhibitor was measured using western blot and qRT-PCR. *p < 0.05, **p < 0.01, ***p < 0.001

We further explored the mechanism underlying the M2 macrophage-inducing effects of exosomal miR-301a-3p from ESCC cells. Data from Targetscan showed that the 3′UTR sequence of *PTEN* might possess latent binding sites with the miR-301a-3p sequence at positions 412–418, suggesting that miR-301a-3p might target *PTEN* to induce M2 polarization (Fig. 6a). Subsequently, we confirmed the binding of miR-301a-3p to *PTEN* using a dual-luciferase reporter assay, which demonstrated a significant inhibition of luciferase activity in the PTEN-3'-UTR WT construct in miR-301a-3p mimic-transfected 239 T cells. In contrast, no effect was observed when PTEN-3'-UTR MUT was used, proving that *PTEN* could be directly targeted by miR-301a-3p (Fig. 6b).

In addition, qRT-PCR results from 20 patients demonstrated lower *PTEN* expression in ESCC tissues than in corresponding normal adjacent esophageal mucosa tissues (Fig. 6c). Through correlation analysis, we found a negative association between miR-301a-3p levels and *PTEN* levels, further validating the direct targeting of *PTEN* by miR-301a-3p (Fig. 6d). Clinicopathological analysis results suggested that the *PTEN* level was inversely related to tumor diameter (p = 0.020), tumor invasion (p = 0.004), TNM stage (p = 0.032), and lymph node metastasis (p = 0.017), whereas it showed no association with age and gender (Table 3), indicating that PTEN was a tumor suppressor of ESCC.

**Table 3:** Relationship between PTEN expression and clinicopathologic characteristics of 20 patients with ESCC

Variables	Total	PTEN expression		
		Low	High	*P* value
Gender				
Male	13	8	5	0.491
Female	7	2	5	
Age (years)				
< 60	4	3	1	0.173
> 60	16	7	9	
Tumor diameter (cm)				
< 3	3	1	2	0.020*
> 3	17	9	8	
T stage				
T1 + T2	8	3	5	0.004**
T3 + T4	12	7	5	
Lymph node metastasis				
N^−^	10	4	6	0.017*
N^+^	10	6	4	
TNM stage				
I + II	11	4	7	0.032*
III + IV	9	6	3	

The PI3K/AKT signaling pathway participates in macrophage polarization. To further explore whether this pathway mediates the macrophage polarization induced by ESCC-derived exosomes, we examined the expression of PI3K and AKT in macrophages treated with PBS or ESCC-derived exosomes using western blot and qRT-PCR. We found that PI3K and AKT levels were significantly upregulated in macrophages incubated with ESCC-derived exosomes (Fig. 6e and f). Moreover, the expression of PTEN, which inhibits the PI3K/AKT signaling pathway, was down-regulated in macrophages incubated with ESCC-derived exosomes (Fig. 6e and f), suggesting that ESCC-derived exosomes induced M2 macrophage polarization via the inhibition of PTEN and activation of the PI3K/AKT signaling pathway, and then promotes angiogenesis.

Recent studies have confirmed that TAMs can secret pro-angiogenic factors such as VEGFA and MMP9 and thereby promote tumor angiogenesis. In order to explore whether macrophages incubated with ESCC-derived exosomes can promote angiogenesis via the upregulation of VEGFA and MMP9, the expression of these factors in macrophages incubated with PBS or ESCC-derived exosomes was examined using western blot and qRT-PCR. VEGFA and MMP9 levels were significantly elevated in macrophages incubated with ESCC-derived exosomes (Fig. 6g and h). ELISA demonstrated that VEGFA and MMP9 expression in the supernatant of macrophages incubated with ESCC-derived exosomes was significantly higher than that in the supernatant of macrophages incubated with PBS (Fig. 6i). Hence, exosomes from ESCC cells can induce M2 macrophage polarization by inhibiting *PTEN* and activating PI3K/AKT signaling and then promote angiogenesis by secreting VEGFA and MMP9.

To further examine the role of PI3K/AKT signaling in the upregulation of VEGFA and MMP9, macrophages incubated with ESCC-derived exosomes were treated with LY294002, a PI3K inhibitor. Western blot and qRT-PCR demonstrated that LY294002 treatment attenuated the upregulation of PI3K, AKT, VEGFA, and MMP9 induced by exosomes secreted from ESCC cells (Fig. 6j and k).

Moreover, *PTEN* expression was lower and PI3K, AKT, VEGFA, and MMP9 expression was higher in macrophages co-cultured with exosomes from miR-301a-3p mimic-transfected EC9706 cells than in those co-cultured with exosomes from miR-NC-transfected EC9706 cells. However, the opposite trend was observed when the miR-301a-3p inhibitor was used for transfection (Fig. 6l and m). All the results above demonstrated that ESCC-derived exosomal miR-301a-3p induces M2 macrophage polarization via the inhibition of PTEN and activation of the PI3K/AKT signaling pathway, and then promotes angiogenesis via the secretion VEGFA and MMP9.

## Discussion

Angiogenesis is crucial for maintaining survival and progression in solid tumors. Therefore, angiogenesis inhibition via targeted drug treatment has become a prospective therapeutic strategy for various malignant tumors. Several antiangiogenic drugs, such as bevacizumab and Zaltrap, have received approval from the Food and Drug Administration (FDA) as first-line treatment agents for glioblastoma, colorectal cancer, and other cancers and have provided encouraging outcomes [18, 19]. However, ramucirumab remains the only FDA-approved antiangiogenic drug which is highly effective against esophageal cancer. Moreover, most clinical trials have focused on ECA and esophageal gastric junction cancer, which are common in western populations. Data on ESCC, which is very prevalent in China, is relatively scarce and warrants more exploration [20, 21]. Therefore, investigating the molecular mechanism underlying tumor angiogenesis in ESCC may help clarify the complex angiogenic process in the TME of ESCC, enabling the development of effective antiangiogenic and anti-cancer drugs for patients with ESCC in the future.

Increasing evidence has shown that exosomes, which are natural endogenous vesicles, can be used as anti-cancer drug carriers. Exosomes play important roles in tumorigenesis, cancer progression, metastasis, drug resistance, and angiogenesis owing to the miRNA they carry [22]. An immune tolerance environment, which promotes tumor progression, can be created by the exosome-mediated crosstalk of tumor cells with other cells in the TME. Exosomes from pancreatic tumor cells have been found to induce apoptosis in T lymphocytes by activating p38 MAPK and causing endoplasmic reticulum stress [23]. The cytotoxicity of natural killer cells has been found to decrease after long-term incubation with oral cancer cell‑derived exosomes [24]. In our study, we discovered that exosomes secreted by ESCC cells induce M2 macrophage polarization via the PTEN/PI3K/AKT signaling pathway. Our findings indicated that exosomes are essential messengers mediating intercellular communication between tumor cells and surrounding stromal cells in the TME.

VEGFA is commonly recognized as the most effective angiogenesis-promoting factor, and it can activate a variety of downstream signaling pathways in endothelial cells, consequently exerting a positive effect on the proliferation and migration of endothelial cells [25]. Increased serum levels of VEGF and EGF have been confirmed to show a strong correlation with a poor prognosis in ESCC patients [26]. MMP9, a core member of the MMP family, can degrade gelatin, fibronectin, and other protein components. Therefore, it assists in the proliferation and migration of endothelial cells by degrading the basement membrane and extracellular matrix [27]. Our study demonstrated that macrophages facilitate proliferation, migration, invasion, and tube formation in TECs by secreting VEGFA and MMP9, confirming the promoting effect of TAMs on tumor angiogenesis.

miR-301a-3p, belonging to the mir-130/301 family, is a multifunctional miRNA that promotes tumor progression, regulates inflammatory responses, and mediates drug resistance [28–30]. miR-301a-3p can promote tumor proliferation and metastasis in several malignancies, including esophageal, pancreatic, and colorectal cancer [31–33]. Nevertheless, the mechanism via which miR-301a-3p contributes to ESCC progression has been unclear, and research on how miR-301a-3p affects angiogenesis is extremely scarce. Our findings confirmed miR-301a-3p upregulation in ESCC tissues and cells and showed that the level of this miRNA was positively correlated with TNM stage, tumor invasion, and lymph node metastasis. Our findings thus pointed to a potential regulatory role of this miRNA in the pro-angiogenic switch in macrophages via the PTEN/PI3K/AKT pathway.

Previous studies on angiogenesis have mainly focused on the direct effect of cancer cells or other cells in the TME on angiogenesis, while the mechanism by which cancer cells indirectly promote angiogenesis through effects on the TME remained unclear. Research has suggested that exosomal miR-155-5p from melanoma cells induces the transformation of normal fibroblast into cancer-associated fibroblasts (CAFs), thereby promoting angiogenesis, indicating that cancer cells can indirectly affect angiogenesis via CAFs in the TME [34]. However, how cancer cells exert indirect effects on angiogenesis by affecting TAMs remained to be explored. In particular, the mechanism by which cancer cells use exosomal miRNA to indirect affect angiogenesis via the crosstalk with TAMs had not been reported. Our study demonstrates that ESCC-derived exosomal miR-301a-3p can induce M2 polarization via the PTEN/PI3K/AKT signaling pathway and then indirectly promote angiogenesis.

However, there are some limitations to our study. First, the sample size used for expression analysis in tumor tissues was relatively limited. Hence, further research with a larger sample size is warranted. Second, we were unable to perform in vivo assays owing to uncontrollable external circumstances. We will validate our results with such experiments in our future studies.

## Conclusions

Exosomal miR-301a-3p secreted from ESCC cells causes M2 macrophage polarization via the inhibition of PTEN expression and activation of the PI3K /AKT signaling pathway and then promotes angiogenesis via the secretion of the angiogenic factors VEGFA and MMP9. Our study reveals a novel mechanism underlying the crosstalk between ESCC cells and TAMs, which triggers the pro-angiogenic switch of macrophages. Our findings lay the foundation for formulating new strategies and developing effective antiangiogenic drugs for ESCC treatment. Other biological molecules carried by ESCC cell-derived exosomes may also be involved in the pro-angiogenic switch of TAMs, and these warrant further study.

## Data Availability

All datasets obtained and analyzed during this study are available from the corresponding author on reasonable request.
